# Clinical Efficacy and Safety of Propranolol in the Prevention and Treatment of Retinopathy of Prematurity: A Meta-Analysis of Randomized Controlled Trials

**DOI:** 10.3389/fped.2021.631673

**Published:** 2021-02-10

**Authors:** Haibo B. Kong, Guoyuan Y. Zheng, Baomei M. He, Ying Zhang, Qin Zhou

**Affiliations:** ^1^Department of Pediatrics, Zhejiang Provincial People's Hospital, People's Hospital of Hangzhou Medical College, Hangzhou, China; ^2^Department of Neuroelectrophysiology, Zhejiang Provincial People's Hospital, People's Hospital of Hangzhou Medical College, Hangzhou, China

**Keywords:** retinopathy of pre-maturity, propranolol, clinical efficacy, safety, meta-analysis

## Abstract

**Objective:** To perform a meta-analysis of randomized controlled trials verifying clinical efficacy and safety of propranolol in pre-term newborns with retinopathy of prematurity (ROP).

**Methods:** We searched the literature databases (Pubmed, Embase, The Cochrane Library, Web of Science, CNKI, WanFang, VIP, CBM) for publications before August 10, 2020, and the World Health Organization's International Clinical Trials Registry and ClinicalTrials.gov for ongoing trials. Randomized controlled trials (RCTs) of propranolol for the prevention or treatment of ROP were included. The quality of the included studies was primarily assessed by the RCT tool of the Cochrane Collaboration. The included studies were quantified using a meta-analysis of relative risk (RR) estimated with a random effect model.

**Results:** Our original search identified 171 articles, and five studies met our criteria. A meta-analysis was performed that showed that infants orally treated with propranolol had a decreased risk of disease progression: stage progression had an RR = 0.65 [95% confidence interval (CI), 0.47–0.88]), plus disease had an RR = 0.43 [95% CI, 0.22–0.82]. The demands for additional treatments had similar protective results: laser photocoagulations had an RR = 0.55 [95% CI, 0.35–0.86]), and intravitreal injection of anti-vascular endothelial growth factor had an RR = 0.45 [95% CI, 0.22–0.90]). The oral administration of propranolol was associated with an increased risk of adverse events (RR = 2.01 [95% CI, 1.02–3.97]). High-risk adverse events included bradycardia, hypotension, not gaining enough weight, bronchospasm, hypoglycemia, apnea, and increasing ventilator need. Subgroup analysis of ROP phases and stages found that the risk in stage 2 ROP of the second phase and the individual risk factors (stage progression, RR = 0.42 [95% CI, 0.27–0.65]; plus disease, RR = 0.40 [95% CI, 0.17–0.93]; laser photocoagulation, RR = 0.31 [95% CI, 0.14–0.68]) have statistically significant differences compared with other phases and stages.

**Conclusions:** Pre-term newborns with ROP, especially in stage 2 ROP of the second phase, who were orally given propranolol have a reduced risk of disease progression and demand for additional treatments, but the safety needs more attention.

## Introduction

Retinopathy of pre-maturity (ROP) is a complex eye disease involving immature development, oxygen, inflammation, and other factors, and it leads to microvascular lesions, resulting in new blood vessels and ultimately leading to retinal detachment (1). Globally, it is estimated that more than 20,000 babies are blinded by ROP each year, and another 12,300 have mild-to-moderate visual impairment (2). In addition to visual loss, ROP can lead to a wide range of other visual impairments, including reduced contrast sensitivity, visual field deficits, color vision deficits, strabismus, and refractive errors (3). With the rapid development and application globally of neonatal intensive care technology, pre-term infants' survival rate has been significantly increased, and the incidence of common complications, such as ROP, has also increased accordingly (4).

ROP is a biphasic disease with two phases, including blood vessel growth arrest that leads to ischemia and uncontrolled proliferation of blood vessels (5). Currently, the treatment is mainly focused on the second phase of ROP, including laser photocoagulation and intraocular injection of vascular endothelial growth factor (VEGF)-neutralizing antibodies (6). However, despite these treatments, an increasing number of clinical studies report that laser photocoagulation (7) and intravitreal injection of anti-VEGF therapy (8, 9) do have some limitations, which can cause several side effects. Therefore, it is necessary to search for new therapeutic alternatives that avoid or reduce complications or sequelae due to laser photocoagulation and intravitreal injection of anti-VEGF therapy.

In 2008, French scholars accidentally discovered that propranolol could effectively control infantile hemangioma proliferation and promote its regression (10). Since then, many scholars from a variety of countries have studied propranolol. In 2010, the first study suggested that propranolol has a potential efficacy on ROP (11). In 2015, propranolol was suggested for early prevention of ROP and treatment of existing ROP in pre-term neonates (12). However, there is no consensus about either the benefit or the concerns of using propranolol for ROP treatment. Several randomized controlled trials (RCTs) have recently been published. This study aimed to conduct a meta-analysis on the clinical efficacy and safety of propranolol to prevent and treat ROP.

## Methods

### Protocol and Registration

According to a pre-published protocol on PROSPERO (CRD42020204510), we performed this meta-analysis following the methodology suggested by Q Zhou et al., the Cochrane Handbook for Systematic Reviews of Interventions, and the PRISMA statement.

### Eligibility Criteria

The inclusion criteria were as follows: (1) RCTs were considered for inclusion irrespective of blinding, publication status, or sample size; (2) the pre-term patients had a high risk of developing ROP or had been diagnosed with ROP by the international classification of ROP (13) regardless of their underlying disease; (3) the experimental intervention was propranolol, independent of propranolol dose, manner of administration, and duration of treatment; and (4) the control intervention was either a placebo or no treatment. (5) Primary outcomes were disease progression (stage progression and plus disease), the demands for additional treatments (laser photocoagulation and intravitreal injection of anti-VEGF), and adverse events.

The exclusion criteria were as follows: (1) duplicate studies and data, (2) studies with incomplete or missing data or studies that were only an abstract with no full text, (3) studies not reported in Chinese or English.

### Data Sources and Searches

We searched the following databases for relevant English language literature: PubMed, EMBASE, the Cochrane Library, and Web of Science and Chinese language literature: CNKI, WanFang, VIP, and CBM. The search string was built as follows (such as Pubmed): (“retrolental fibroplasia”[Title/Abstract] OR (“retinopathy”[Title/Abstract] AND “prematur^*^”[Title/Abstract]) OR “Retinopathy of Prematurity”[MeSH Terms]) AND ((“Propranolol”[Title/Abstract] OR “Inderal”[Title/Abstract] OR “Avlocardyl”[Title/Abstract] OR “Dexpropranolol”[Title/Abstract] OR “Dociton”[Title/Abstract] OR “anaprilin^*^”[Title/Abstract] OR “Betadren”[Title/Abstract] OR “ay 20694”[Title/Abstract] OR “obsidan”[Title/Abstract] OR “obzidan”[Title/Abstract] OR “propanolol”[Title/Abstract]) OR “Propranolol”[MeSH Terms]). References of the identified studies were screened to identify further relevant trials by two reviewers (HB Kong and GY Zheng). In addition, we searched the World Health Organization's International Clinical Trials Registry and ClinicalTrials.gov for ongoing trials. The search was last updated on August 10, 2020 (The retrieval strategy is detailed in Supplement 1 in Supplementary Material).

### Data Extraction and Quality Assessment

#### Data Extraction

Two authors (HB Kong and GY Zheng) independently extracted the study data. Any disagreement was resolved by discussion until consensus was reached or by consulting a third author (Q Zhou). The following data were extracted: author, year of publication, county, original inclusion and exclusion criteria, the total number of people included in the study, doses of propranolol and time of application, numbers of progression to higher ROP stage, numbers of progression with plus disease, numbers of treatment with laser photocoagulation, numbers of treatment with intravitreal injection of anti-VEGF, and adverse events.

#### Quality Assessment

Two reviewers (HB Kong and GY Zheng) independently assessed the quality of the selected studies according to the Cochrane Collaboration's tool for RCTs. Papers were evaluated in three categories: low risk of bias, unclear bias, and high risk of bias. The following characteristics were evaluated: random sequence generation (selection bias), allocation concealment (selection bias), blinding of participants and personnel (performance bias), incomplete outcome data (attrition bias), selective reporting (reporting bias), and other biases; results were graphed and assessed using Review Manager 5.3. In addition, the studies were graded by quality assessment methods (14) as low, high, or moderate quality to facilitate the subgroup analysis and sensitivity analysis.

### Data Synthesis and Analysis

#### Meta-Analysis

Relative risks (RR) and the random effect model (weighted by the Mantel-Haenszel) were used. Results were assessed using forest plots and presented as RRs for the primary outcomes. Significant differences (test of interaction *p* < 0.05) were considered statistically significant. Statistical analyses were conducted using the Review Manager software (Review Manager 5.3, Cochrane Collaboration, Nordic Cochrane Centre, London, United Kingdom).

#### Assessment of Heterogeneity

Between-study heterogeneity was assessed using the τ^2^, χ^2^ (Cochran Q), and *I*^2^ statistics. Consistent with the Cochrane handbook, the *I*^2^ was interpreted as non-important (<30%), moderate (30–60%), and substantial (>60%). The heterogeneity was statistically significant (test of interaction *p* ≤ 0.10 and *I*^2^ > 50%). Clinical heterogeneity was explored by conducting explorative subgroup analysis or sensitivity analysis.

#### Subgroup Analysis

We conducted subgroup analysis to determine whether substantial heterogeneity or clinical significance existed between trials. We performed subgroup analyses for primary outcomes: (1) ROP phase and stage starting to prevent or treat and (2) propranolol dose. Statistically significant subgroup differences (test of interaction *P* < 0.05) could provide evidence of an intervention effect within the subgroup.

#### Sensitivity Analysis

Sensitivity analyses were performed to determine heterogeneity or stability using the meta-analysis results by excluding different research quality (low quality), transforming different inclusion criteria (exclude the first phase ROP study), or shifting different effect models (fixed-effect model).

#### Publication Bias

If there were 10 or more studies in the meta-analysis, we would investigate reporting biases (such as publication bias) using funnel plots.

## Results

### Study Selection

**Figure 1:**
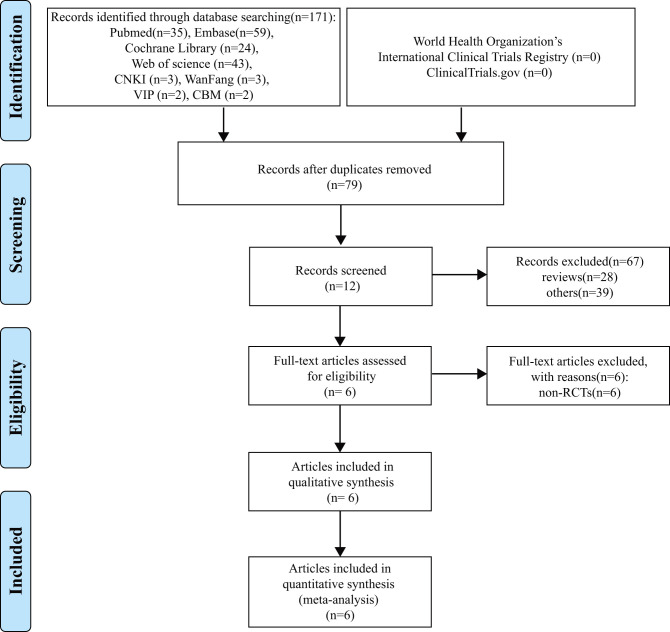
Systematic reviews and meta-analyses (PRISMA) flowchart of study.

Our search strategy identified 171 papers and included 161 papers in English (Pubmed 35, Embase 59, Cochrane Library 24, Web of Science 43) and 10 papers in Chinese (CNKI 3, WanFang 3, VIP 2, CBM 2). After the removal of duplicates and eliminating apparently unrelated studies by reading the titles and abstracts, 12 records remained. Six records were excluded based on full text, and the remaining six records were included. Among these articles, the study Ozturk and Korkmaz (15) was a supplement to Korkmaz et al. (16) and their data complement each other. Finally, five RCTs (six articles) with 445 patients were included in the quantitative synthesis (Figure 1).

### Characteristics of the Included Studies

Detailed characteristics of the five included trials are presented in Table 1. The year of publication ranged from 2013 to 2018. Four trials were reporting in English and one in Chinese. One trial was published as a letter only. There were three single-center and two multicenter trials (Table 1).

**Table 1:** Characteristics of included randomized trials.

**References**	**Country**	**Inclusion criteria**	**Exclusion criteria**	**Phase and stage**	**Intervention group**							**Control group**				**Outcome**
					**Sample size**	**Gestational age (weeks)**	**Male (%)**	**Intervention**	**Dose**	**Way**	**Duration**	**Sample size**	**Gestational age (weeks)**	**Male (%)**	**Control**	
Filippi et al. (17)	Italy	GA < 32 w; Stage 2 ROP without plus in zone II.	Congenital or acquired cardiovascular anomalies, renal failure or cerebral hemorrhage, ROP in zone I, more advanced stage than Stage 2 without plus in zone II.	Second phase-stage 2	26	26.5 ± 2.2	17 (65)	Propranolol	0.5 or 0.25 mg/kg/q6h	Oral	Until complete retinal vascularization, not more than 90 days.	26	26.2 ± 1.7	15 (58)	Standard treatment alone	1. Progression to higher stage; 2. with plus; 3. Treatment with laser photocoagulation; 4. Treatment with intravitreal injection of anti-VEGF; 5. Adverse events.
Makhoul et al. (18)	Israel	24 w < GA < 28 w, BW < 1,500 g, Stage 1 (zone I), Stage 2 or higher (zones I or II), and/or plus disease.	None.	Second phase-stage 1 or higher	10	None	None	Propranolol	0.16 mg/kg/q8 h to 0.67 mg/kg/q8h	Oral	Given for 4 weeks or until ROP resolution or hospital discharge.	10	None	None	Placebo (sucrose 5%)	1. Progression to higher stage; 2. Treatment with laser photocoagulation; 3. Treatment with intravitreal injection of anti-VEGF; 4. Adverse events.
Korkmaz et al. (16)	Turkey	GA < 32 w, BW < 1,500 g, Stage 0, 1, 2 ROP.	Cardiovascular anomaly, renal failure, apnea, hypoglycemia, bradycardia, not take medicine, parents' request, not gain sufficient weight.	Second phase-stage 0,1,2	89	28.3 ± 2.03	None	Propranolol	0.5 mg/kg/q6h	Oral	Until complete retinal vascularization.	91	28.6 ± 1.82	None	Physiological saline	1. Treatment with laser photocoagulation; 2. Treatment with intravitreal injection of anti-VEGF; 3. Adverse events.
Sanghvi et al. (19)	India	26 w < GA < 32 w, ≤7 days old.	Recurrent episodes of bradycardia, atrioventricular blocks, hypotension, refractory hypoglycaemia and major congenital malformations.	First phase	55	29.54 ± 1.69	24 (44)	Propranolol	0.5 mg/kg/q12h	Oral	Utill a corrected gestational age of 37 weeks or complete retinal vascularisation.	54	29.12 ± 1.74	29 (54)	Calcium carbonate	1. progression to higher stage; 2. with plus; 3. treatment with laser photocoagulation; 4. treatment with intravitreal injection of anti-VEGF; 5. Adverse events.
Ozturk and Korkmaz, (15) [Supplement the Korkmaz et al. (16) data]	Turkey	GA < 32 w, BW < 1,500 g, Stage 0, 1, 2 ROP.	Cardiovascular anomaly, renal failure, apnea, hypoglycemia, bradycardia, not taken their medicine, not gain sufficient weight, and their parents' request.	Second phase-stage 0,1,2	58	28.4 ± 1.23	32 (55)	Propranolol	2 mg/kg/d	Oral	Until complete retinal vascularization.	68	28.6 ± 1.54	39 (57)	Physiological saline	1. Progression to higher stage; 2. With plus;3. Adverse events.
Sun et al. (20)	China	GA < 32 w, Stage 2 ROP without plus in zone II or III.	Genetic metabolic diseases, congenital dysplasia, congenital heart disease, severe chronic lung disease, septicemia and renal failure, severe bradycardia and hypotension within 0.5–1 h after taking propranolol, and their parents' request.	Second phase-stage 2	41	29.9 ± 1.8	28 (68)	Propranolol	0.25 mg/kg/q12h	Oral	Until complete retinal vascularization, or hospital discharge, not more than 30 days.	43	30.1 ± 1.7	27 (63)	Physiological saline	1. Progression to higher stage; 2. Treatment with laser photocoagulation; 3. Treatment with intravitreal injection of anti-VEGF; 4. Adverse events.

### Bias Risk Assessment

**Figure 2:**
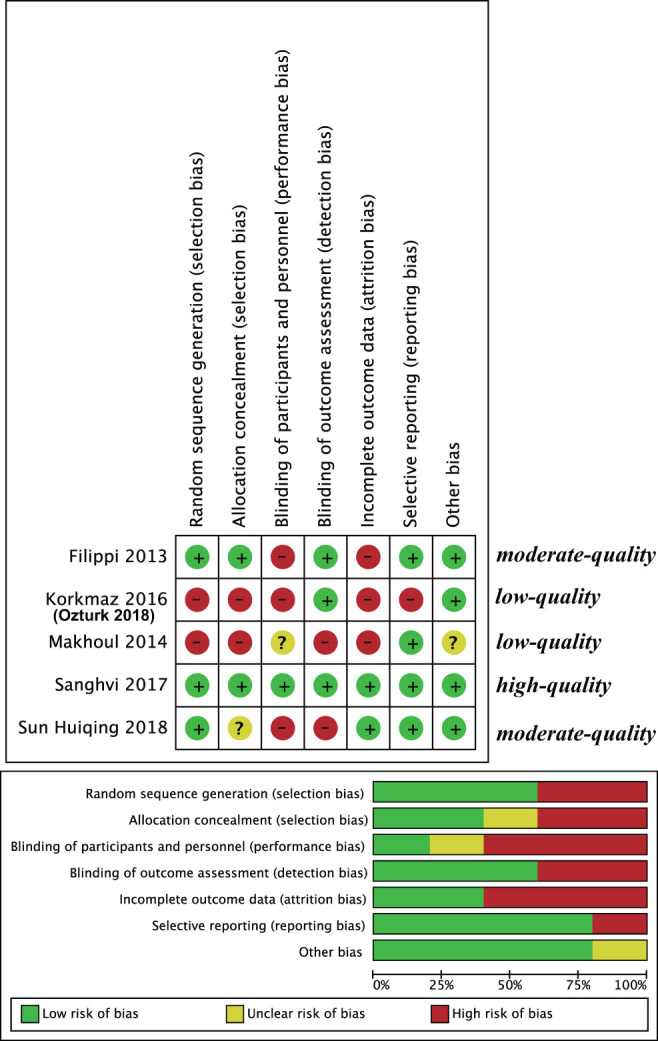
Risk of bias assessment.

The risk of bias for the included RCTs was assessed using the Cochrane Risk of Bias tool. Random sequence generation, allocation concealment, blinding participants and personnel, blinding of outcome assessment, incomplete outcome data, selective reporting, and other biases (defined by the Cochrane tools as including bias due to problems not covered elsewhere, such as a particular trial design, alleged fraud, or other problems) were evaluated. In addition, quality assessment methods were performed as follows: (1) If randomization or allocation concealment was considered to have a high risk of bias without considering the risks of other items, the trial quality was considered to be low; (2) when both randomization and allocation concealment were assessed to have a low risk of bias and all other items were assessed to have a low or unclear risk of bias, the trial quality was considered to be high; (3) tests that did not meet high- or low-quality standards were considered to be moderate quality (14). Sanghvi et al. (19) was high quality, Filippi et al. (17) and Sun et al. (20) were moderate quality, and Korkmaz et al. (16) [Ozturk and Korkmaz (15)] and Makhoul et al. (18) were low quality (Figure 2).

### Meta-Analysis

#### Clinical Efficacy

**Figure 3:**
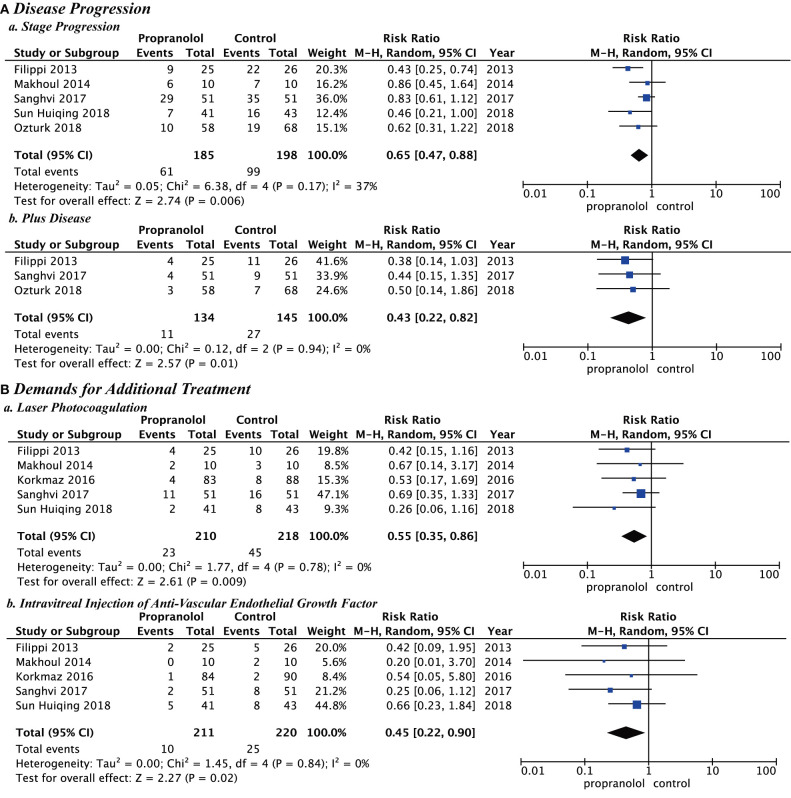
Clinical efficacy of propranolol oral administration in the prevention and treatment of retinopathy of prematurity based on disease progression and the demands for additional treatment. **(A)** Disease progression and **(B)** Demands for additional treatment.

In all studies, the main outcomes of clinical efficacy were prevention of disease progression and reduction of additional treatments. Disease progression included stage progression and plus disease. The demands for additional treatment included laser photocoagulation and intravitreal injection of anti-VEGF. The propranolol group was significantly better than the control group for stage progression (RR = 0.65; *P* = *0*.006; *I*^2^ = 37%), plus disease (RR = 0.43; *P* = *0*.01; *I*^2^ = 0%), laser photocoagulation (RR = 0.55; *P* = *0*.009; *I*^2^ = 0%), and intravitreal injection of anti-VEGF (RR = 0.45; *P* = *0*.02; *I*^2^ = 0%) (Figure 3).

#### Safety

**Figure 4:**
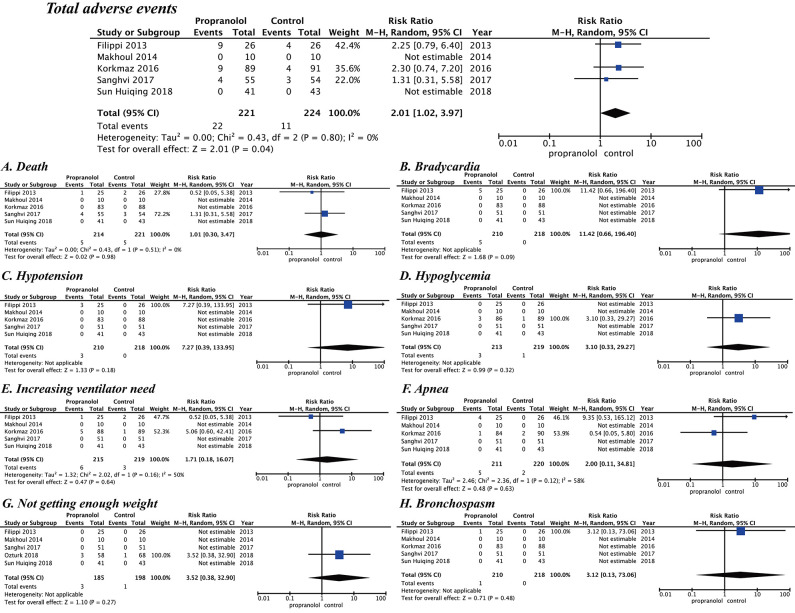
Safety of propranolol oral administration in the prevention and treatment of retinopathy of prematurity based on adverse events. **(A)** Death, **(B)** Bradycardia, **(C)** Hypotension, **(D)** Hypoglycemia, **(E)** Increasing ventilator need, **(F)** Apnea, **(G)** Not getting enough weight, and **(H)** Bronchospasm.

For the five included studies, those from Filippi et al. (17), Korkmaz et al. (16) [Ozturk and Korkmaz (15)], and Sanghvi et al. (19) report several adverse events, but those from Makoul et al. (18) and Sun et al. (20) report no adverse events. Details of these adverse events are shown in Table 2. A meta-analysis of the five trials evaluated the safety of propranolol in the course of treatment. Propranolol was associated with an increased RR for overall adverse events (RR = 2.01 [95% confidence interval (CI), 1.02 to 3.97]). Sun et al. (20) and Makoul et al. (18) had no adverse events during propranolol treatment. The other studies report adverse events, including death (RR = 1.01 [95% CI, 0.30–3.47]), bradycardia (RR = 11.42 [95% CI, 0.66–196.40]), hypotension (RR = 7.27 [95% CI, 0.39–133.95]), hypoglycemia (RR = 3.10 [95% CI, 0.33–29.27]), increasing ventilator need (RR = 1.71 [95% CI, 0.18–16.07]), apnea (RR = 2.00 [95% CI, 0.11–34.81]), not gaining enough weight (RR = 3.52 [95% CI, 0.38 to 32.90]), and bronchospasm (RR = 3.12 [95% CI, 0.13–73.06]) (Figure 4).

**Table 2:** Adverse events in included Studies.

**References**	**Intervention group (22/221)**	**Control group (11/224)**
Filippi et al. (17)	1 case of death, 1 case of increasing ventilator need, 3 cases of serial apnea, bradycardia, and hypotension, 1 case of severe apnea and bradycardia, 1 case of bradycardia with H1N1 infection, 1 case of bronchospasm, and 1 case of unknown reason (unreported).	2 cases of death and 2 cases of increasing ventilator need.
Makhoul et al. (18)	None.	None.
Korkmaz et al. (16) [Ozturk and Korkmaz (15)]	1 case of apnea, 2 cases of increasing ventilator need, 3 cases of hypoglycemia and increasing ventilator need, and 3 cases of not getting enough weight.	2 cases of apnea, 1 case of increasing ventilator need and hypoglycemia, and 1 case of not getting enough weight.
Sanghvi et al. (19)	4 cases of death.	3 cases of death.
Sun et al. (20)	None.	None.

### Subgroup Analysis

**Figure 5:**
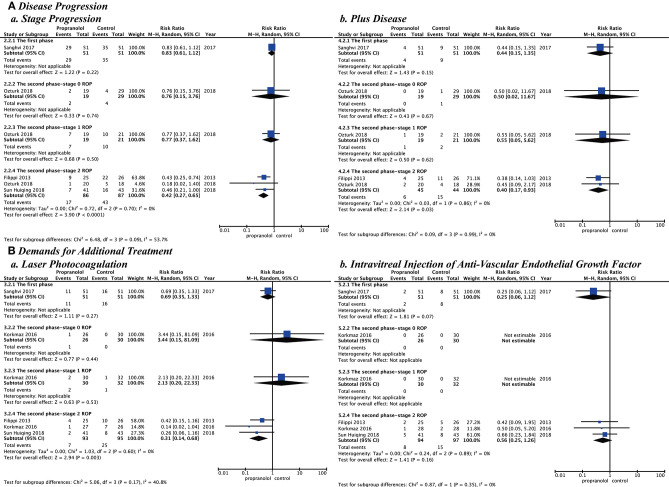
Subgroup analysis with different retinopathy of prematurity phase and stage when starting to prevent or treat. **(A)** Disease progression and **(B)** Demands for additional treatment.

**Figure 6:**
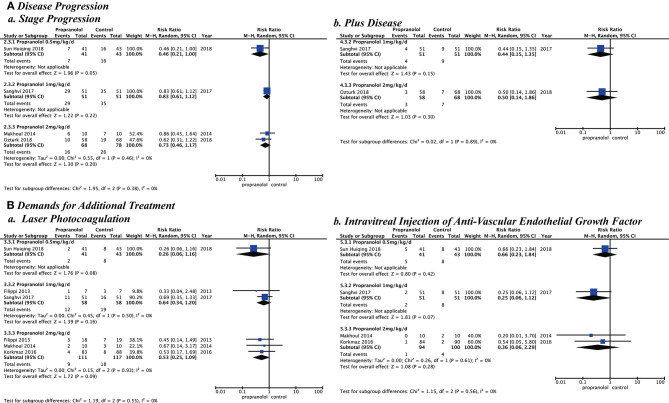
Subgroup analysis with different doses of propranolol oral administration when starting to prevent or treat. **(A)** Disease progression and **(B)** Demands for additional treatment.

After assessment of heterogeneity, the heterogeneity among studies was not statistically significant (*p* > 0.10 and *I*^2^ < 50%), but the timing and dosage of propranolol are clinical concerns. Different ROP phases and stages starting to prevent or treat and different propranolol doses would be subgroup analyzed. Subgroup analysis showed the following risks in Stage 2 ROP of the second phase: (stage progression, RR = 0.42 [95% CI, 0.27–0.65]; plus disease, RR = 0.40 [95% CI, 0.17–0.93]; laser photocoagulation, RR = 0.31 [95% CI, 0.14–0.68]). Stage 2 ROP of the second phase had statistically significant differences for these variables although the other phases and stages did not. Different starting doses of oral propranolol to prevent or treat ROP showed that the risk in the low dose (0.5 mg/kg/d, stage progression, RR = 0.46 [95% CI, 0.21–1.00]; laser photocoagulation, RR = 0.26 [95% CI, 0.06–1.16]; intravitreal injection of anti-VEGF (RR = 0.66 [95% CI, 0.23–1.84]) or high dose (2 mg/kg/d, stage progression, RR = 0.73 [95% CI, 0.46–1.17]; plus disease, RR = 0.50 [95% CI, 0.14–1.86]; laser photocoagulation, RR = 0.49 [95% CI, 0.21–1.13]; intravitreal injection of anti-VEGF (RR = 0.36 [95% CI, 0.06–2.29]) had no statistically significant difference (Figures 5, 6).

### Sensitivity Analysis

**Figure 7:**
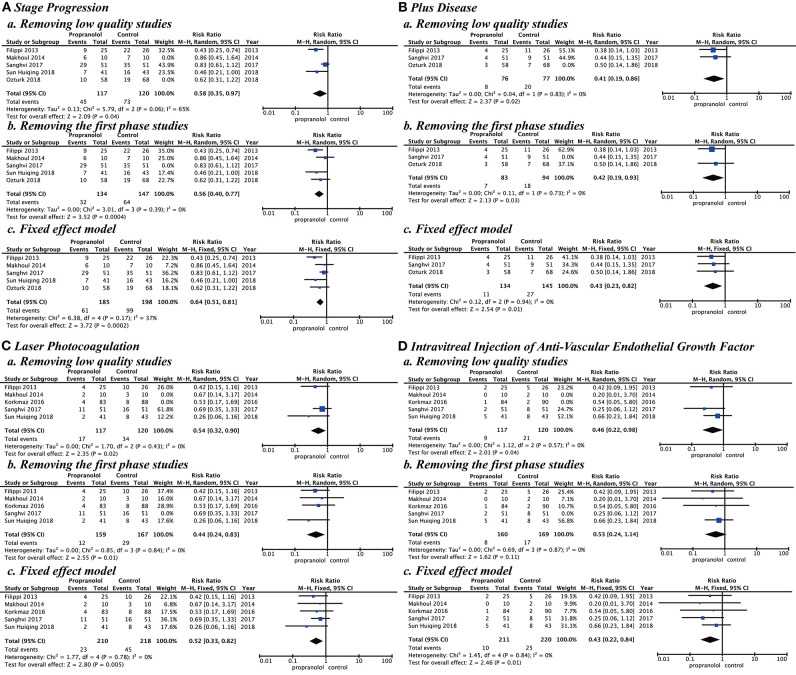
Sensitivity analysis by research quality, inclusion criteria, and effect model. **(A)** Stage progression, **(B)** Plus disease, **(C)** Laser photocoagulation, and **(D)** Intravitreal injection of anti-vascular endothelial growth factor.

Sensitivity analysis by research quality, inclusion criteria, and effect model was performed. In the research quality, when low-quality studies (15, 16, 18) were removed, the results showed that the propranolol group had a significantly better effect than the control group with stage progression (RR = 0.58 [95% CI, 0.35–0.97]), plus disease (RR = 0.41, [95% CI, 0.19–0.86]), laser photocoagulation (RR = 0.54 [95% CI, 0.32–0.90]), and intravitreal injection of anti-VEGF (RR = 0.46 [95% CI, 0.22–0.98]). For the inclusion criteria study, when we removed the study (19) that used propranolol in the first phase of ROP, our results showed that the propranolol group had a significantly better effect than the control group with stage progression (RR = 0.56 [95% CI, 0.40–0.77]), plus disease (RR = 0.42 [95% CI, 0.19–0.93]), and laser photocoagulation (RR = 0.44 [95% CI, 0.24–0.83]). When we used the fixed-effect model, the results showed that the risk in the propranolol group was significantly better than in the control group with stage progression (RR = 0.64 [95% CI, 0.51–0.81]), plus disease (RR = 0.43 [95% CI, 0.23–0.82]), laser photocoagulation (RR = 0.52 [95% CI, 0.33–0.82]), and intravitreal injection of anti-VEGF (RR = 0.43 [95% CI, 0.22–0.84]) (Figure 7).

### Publication Bias

All outcome indicators were analyzed in <10 studies, so publication bias was not examined.

## Discussion

According to the five included studies on ROP treatment, we found that the RR of disease progression and the demands for additional treatment were significantly lower compared with that without propranolol. However, we found an increased RR for adverse events compared with those without propranolol. In subgroup analysis, it was found that, when the propranolol is initiated at Stage 2 ROP, the propranolol had the most significant clinical effect in stage progression, plus disease, and laser photocoagulation compared with other phases or stages. In addition, our analysis found no significant difference in clinical effects in different therapeutic doses. The sensitivity analysis found that the research results were stable according to the research quality, inclusion criteria, and effect model changes.

ROP, initially known as retinal fibrosis, is characterized by vasoproliferative retinopathy and primarily affects newborns born at <32 weeks of gestation. Pre-term birth (low gestational age, low birth weight), hyperoxygen supplementation, poor post-partum weight gain, hyperglycemia, low IGF-1 concentration, blood transfusion, and infection are associated with developing ROP (21). ROP's complex pathogenesis occurs in 2 phases. In the first phase, the blood vessel stops growing and leads to ischemia; in the second phase, blood vessel proliferation occurs (5). Neovascularization is related to a variety of growth factors, such as VEGF, platelet-derived factor, interleukin-8, and others. VEGF is a kind of endothelial cell–selective mitogen and is a key factor in a series of continuous reactions causing vascular leakage and angiogenesis (22, 23). Adrenergic receptors, such as beta2- and beta3-adrenal receptors, play an essential role in regulating the VEGF level (24, 25). Propranolol is a non-selective beta-adrenal receptor blocker, which has antagonistic effects on sympathetic excitation and catecholamine and can affect angiogenesis.

Animal experiments confirm that propranolol can reduce the excessive production of VEGF in the hypoxic retina. However, it does not affect the VEGF level in the normal oxygenated retina and serum, suggesting that VEGF's regulation mechanism at the average oxygen level is different from that in the hypoxic state (26). In addition, the administration of propranolol at the first phase (the ischemic phase), when the level of VEGF is too low, is probably risky for more aggressive ROP development. On the contrary, the administration of propranolol at the second phase (the proliferative phase), when the level of VEGF is usually too high, is rational (27). In Sanghvi et al. (19), pre-term newborns with a gestation age between 26 and 32 weeks and <7 days old were included. Propranolol was given starting from 7 days of age during the first phase of ROP. Although no adverse events were reported, its clinical efficacy was uncertain. Therefore, propranolol is effective in ROP theoretically, particularly at Stage 2 ROP of the second phase because it primarily affects the VEGF levels in the hypoxic retina instead of those in the normal retina. Our study particularly validated such a speculation through a subgroup analysis of the starting point of propranolol treatment.

In infants and children, propranolol, a relatively safe and well-tolerated drug in clinical practice for many years, is commonly used to treat heart disease, neonatal hyperthyroidism, and so on (28). However, for infants with unstable conditions, especially premature infants with moderate-to-severe risk of bronchopulmonary dysplasia, septicemia, dyspnea, or incomplete recovery under anesthesia, propranolol can cause adverse events, such as hypotension and bradycardia (29, 30). Our study found an increased relative risk of adverse events over the patients who did not take propranolol. However, our study found no difference in our baseline data, including complications in premature infants. Propranolol administration route, dose, and duration may be related to the adverse events. Our study also found that adverse events are focused primarily on the Filippi et al. (17) and Korkmaz et al. (16) [Ozturk and Korkmaz (15), the same study as Korkmaz et al. (16)] studies. In Filippi's study, newborns who initially experienced adverse events after receiving high-dose propranolol were reassigned into the low-dose group. Therefore, in these two studies, the propranolol dose was mostly 2 mg/kg/d, which was higher than that in most of the other studies. Although high-dose propranolol was also used in Makhoul's study, the dose was gradually increased, and the follow-up time was significantly insufficient. However, the administration route and duration were similar to other studies, so the extremely high drug dose might cause the adverse events. In addition, no significant difference in the clinical effects was found among different therapeutic doses; good clinical efficacy may also be achieved at low doses.

Compared with previous systematic reviews (31, 32), our study has some advantages. First, this study conducted a comprehensive search, including English databases, such as PubMed, Cochrane Library, EMBASE, and Web of Science, and Chinese databases, such as CNKI, CBM, VIP, and WanFang. Second, this study included 5 RCT studies, which was more than previous studies, and we found that Ozturk and Korkmaz (15) is the same study as Korkmaz et al. (16), which supplements the data from Ozturk and Korkmaz (15) study. Third, in this study, the clinical efficacy and safety of propranolol in ROP treatment were evaluated by multiple indicators including disease progression (stage progression and plus disease), the demands for additional treatment (laser photocoagulation and intravitreal injection of anti-VEGF), and adverse events, all of which have been shown on the forest map. Fourth, although the heterogeneity among studies was low, subgroup analysis was conducted considering clinical attention issues, such as treatment timing and treatment dose.

Similarly, there are still some deficiencies in this study. One RCT study (33) reported by a meeting abstract was excluded because the data were not available. Second, there were fewer than 10 studies included in this study, so publication bias could not be evaluated. Third, in this study, long-term adverse events were not analyzed due to insufficient data. Propranolol has been reported to cause memory loss in chickens through the blood–brain barrier (34). VEGF has the function of protecting neurons. It was found that repeated vitreous injection of VEGF antagonist Bevacizumab could induce retinal neuronal apoptosis (35). Whether propranolol causes damage to retinal neurons or the nervous system remains to be further studied. Fourth, we conducted a quantitative meta-analysis mainly based on secondary data, leading to inaccurate results due to insufficient individual patient data. Fifth, all the randomized controls included in this study were through oral administration. Because no RCT was available before now, other administration routes, such as eye drops, could not be analyzed in this study. However, it is demonstrated that propranolol administered with eye drops reaches the retina (36), and explorative trials with propranolol eye microdrops provide prospective encouraging results (27, 37). Therefore, RCTs using propranolol through eye drops might be expected to show better efficacy and safety than oral approaches.

This study drew the following conclusions: Oral administration of propranolol is effective in preventing or treating ROP. Treatment may be the most effective at the start of ROP Stage 2 of the second phase, and low doses (0.5 mg/kg/d) may have the same therapeutic effect as high doses (2 mg/kg/d). Also, the treatment of ROP with propranolol has some potential safety issues. However, due to the lack of current research, caution should be exercised in interpreting these conclusions. We recommend that additional multicenter, high-quality randomized controlled studies are needed.

## Data Availability Statement

The original contributions presented in the study are included in the article/Supplementary Material, further inquiries can be directed to the corresponding author/s

## Author Contributions

HK and QZ designed the research subject. HK and GZ conducted literature retrieval and screening, and BH and YZ provided guidance in statistical analysis. HK wrote the manuscript. QZ critically revised the manuscript. All authors read and approved the final manuscript.

## Conflict of Interest

The authors declare that the research was conducted in the absence of any commercial or financial relationships that could be construed as a potential conflict of interest.
