# Supplementary material for: Clinical Efficacy and Safety of Propranolol in the Prevention and Treatment of Retinopathy of Prematurity: A Meta-Analysis of Randomized Controlled Trials
Source: Front Pediatr. 2021 Feb 10;9:631673. doi: 10.3389/fped.2021.631673 (PMC7902715; doi:10.3389/fped.2021.631673)
Supplement: Supplementary file 1 [file Table_1.DOCX]

Supplementary Material

**Supplement 1**

**Search strategy**

**Pubmed:**

#1 "retrolental fibroplasia"[Title/Abstract]

#2 "retinopathy"[Title/Abstract] AND "prematur*"[Title/Abstract]

#3 "Retinopathy of Prematurity"[MeSH Terms]

#4 #1 OR #2 OR #3

#5 "Propranolol"[Title/Abstract] OR "Inderal"[Title/Abstract] OR "Avlocardyl"[Title/Abstract] OR "Dexpropranolol"[Title/Abstract] OR "Dociton"[Title/Abstract] OR "anaprilin*"[Title/Abstract] OR "Betadren"[Title/Abstract] OR "ay 20694"[Title/Abstract] OR "obsidan"[Title/Abstract] OR "obzidan"[Title/Abstract] OR "propanolol"[Title/Abstract]

#6 "Propranolol"[MeSH Terms]

#7 #5 OR #6

#8 #4 AND #7

**Web of Science:**

#1 TOPIC: (retinopathy and prematur*) OR TOPIC: ("retrolental fibroplasia")

#2 TOPIC: (Propranolol or Inderal or Avlocardyl or Dexpropranolol or Dociton or anaprilin* or Betadren or ay 20694 or obsidan or obzidan or propanolol)

#3 #1 AND #2

**Embase:**

#1 'retrolental fibroplasia':ti,ab,kw OR (retinopathy:ti,ab,kw AND prematur*:ti,ab,kw)

#2 'retrolental fibroplasia'/exp

#3 #1 OR #2

#4 propranolol:ti,ab,kw OR inderal:ti,ab,kw OR avlocardyl:ti,ab,kw OR dexpropranolol:ti,ab,kw OR dociton:ti,ab,kw OR anaprilin*:ti,ab,kw OR betadren:ti,ab,kw OR 'ay 20694':ti,ab,kw OR obsidan:ti,ab,kw OR obzidan:ti,ab,kw OR propanolol:ti,ab,kw

#5 'propranolol'/exp

#6 #4 OR #5

#7 #3 AND #6

**Cochrane Library:**

#1 (retinopathy and prematur*):ti,ab,kw OR (retrolental fibroplasia):ti,ab,kw

#2 MeSH descriptor: [Retinopathy of Prematurity] explode all trees

#3 #1 OR #2

#4 (Propranolol or Inderal or Avlocardyl or Dexpropranolol or Dociton or anaprilin* or Betadren or ay 20694 or obsidan or obzidan or propanolol):ti,ab,kw

#5 MeSH descriptor: [Propranolol] explode all trees

#6 #4 OR #5

#7 #3 AND #6

**CNKI:**

（篇关摘：早产儿视网膜病）AND（（篇关摘：普萘洛尔）OR（篇关摘：心得安））

**WangFang:**

主题:(早产儿视网膜病) AND 主题:(普萘洛尔 OR 心得安)

**VIP:**

(任意字段=早产儿视网膜病 AND (任意字段=普萘洛尔 OR 任意字段=心得安))

**CBM:**

#1 "早产儿视网膜病"[常用字段:智能]

#2 "早产儿视网膜病"[常用字段:智能]

#3 #1 AND #2

#4 "普萘洛尔"[常用字段:智能] OR "心得安"[常用字段:智能]

#5 "普萘洛尔"[不加权:扩展]

#6 #4 OR #5

#7 #3 AND #6
